# The cellular composition of the tumor microenvironment is an important marker for predicting therapeutic efficacy in breast cancer

**DOI:** 10.3389/fimmu.2024.1368687

**Published:** 2024-02-29

**Authors:** Tingyao Dou, Jing Li, Yaochen Zhang, Wanru Pei, Binyue Zhang, Bin Wang, Yanhong Wang, Hongyan Jia

**Affiliations:** ^1^Department of First Clinical Medicine, Shanxi Medical University, Taiyuan, China; ^2^Department of Breast Surgery, First Hospital of Shanxi Medical University, Taiyuan, Shanxi, China; ^3^Department of Microbiology and Immunology, School of Basic Medical Sciences, Shanxi Medical University, Taiyuan, Shanxi, China; ^4^Key Laboratory of Cellular Physiology (Shanxi Medical University), Ministry of Education, Taiyuan, Shanxi, China

**Keywords:** breast cancer, tumor microenvironment, immune cell, treatment mechanism, combination administration

## Abstract

At present, the incidence rate of breast cancer ranks first among new-onset malignant tumors in women. The tumor microenvironment is a hot topic in tumor research. There are abundant cells in the tumor microenvironment that play a protumor or antitumor role in breast cancer. During the treatment of breast cancer, different cells have different influences on the therapeutic response. And after treatment, the cellular composition in the tumor microenvironment will change too. In this review, we summarize the interactions between different cell compositions (such as immune cells, fibroblasts, endothelial cells, and adipocytes) in the tumor microenvironment and the treatment mechanism of breast cancer. We believe that detecting the cellular composition of the tumor microenvironment is able to predict the therapeutic efficacy of treatments for breast cancer and benefit to combination administration of breast cancer.

## 1 Introduction

### 1.1 Breast cancer (BC)

According to the American Cancer Society, breast cancer accounted for 31% of new cancer cases in women in 2023. Among all cancers affecting women, the incidence of breast cancer is highest (1). Since the mid-2000s, the incidence of female BC has slowly increased by approximately 0.5% every year (2). The mortality rate of female BC peaked in 1989 and has since declined to 43%, mainly due to improvements in early detection, diagnosis and treatment. In recent years, the mortality rate of BC has decreased from 2% to 3% per year between 1990 and 2000 to 1% per year between 2011 and 2020. In 2023, deaths due to female BC made up approximately 15% of all deaths from cancer (1). Although significant progress has been made in the treatment of BC, the metastasis and recurrence are still major challenges for us.

Breast cancer is a heterogeneous disease. Clinically, according to the hormone receptor (ER and PR), HER2 (ERBB2) and proliferation marker protein Ki-67 (MKI67) status, BC can be divided into four subtypes: luminal A, luminal B, HER2-positive and triple-negative breast cancer (TNBC) (3). The classification of BC has an notable influence on patient prognosis. Hormone receptor(HR)-positive BC have a better prognosis than the negative. Moreover, HER2-positive BC are more aggressive than the negative. TNBC which does not express ER, PR, or HER2, is the most aggressive type. TNBC has the highest rate of chemotherapy resistance and distant recurrence, especially brain metastasis. Compared with patients with other subtypes, TNBC patients have the worst prognoses, highest recurrence rates, and the most complex treatments (4).

### 1.2 Treatment of breast cancer

Different subtypes of BC have different first-line treatments. HR-positive BC requires endocrine therapy, such as tamoxifen, aromatase inhibitors and abexilide. HER2-positive BC is treated with anti-HER2 therapy (mainly trastuzumab and patuzumab). TNBC is treated with standard chemotherapy and radiotherapy (5). In the comprehensive treatment of BC, radiotherapy is a vital step. The immunogenicity of BC is low, but TNBC has the highest immunogenicity. Patients who treated with programmed death receptor-1 (PD-1) and its ligand, programmed cell death- ligand 1 (PD-L1) inhibitors have significant efficacy. Since then, immunotherapy has completely changed the treatment for solid tumors (6). Immunotherapy has gradually attracted amounts of attention, but its application in treating BC is still limited. Therefore, several new treatment methods for improving the survival rate of breast cancer patients, such as targeted drugs, vaccines for immune cells, Antibody Drug Conjugates (ADC), nanoparticles (albumin, metals, lipids, polymers, micellar nanoparticles) and breast cancer stem cell (BCSC)-based treatment are emerging and being extensively studied (7).

### 1.3 The tumor microenvironment of breast cancer

Recently, it has been reported that tumors are constantly evolving heterogeneous dynamic systems. The tumor microenvironment (TME) is composed of cancer cells, noncancer cells (including immune cells and stromal cells), and the extracellular matrix (ECM) (8, 9). The interaction between cancer cells and the TME plays an essential role in tumor progression and therapeutic efficacy. According to the immune scores, immune regulatory targets and immune cell infiltration degree, tumors are classified into two types: “immune hot” and “immune cold”. Compared with “immune cold” tumors, the effects of therapy on “immune hot” tumors are better after treated with immunosuppressants. Some immunotherapy mechanisms shift the TME from “cold” to “hot” (10). In fact, cells in the TME regulate tumor growth and progression through different mechanisms and take different parts in the treatment of BC. Studying the contribution of the TME in the progression of BC is conducive to identifying biomarkers that can be used to specifically regulate TME. For example, the T cell concentration is related to the pathological complete response (pCR) and overall survival (OS) of BC patients. Patients with a high change in the CD8/FOXP3 ratio (CFR) have better relapse-free survival (RFS) (11). These indicators may serve as useful biomarkers and drug targets for predicting the efficacy of neoadjuvant therapy (NACT) in the future, but the specific role of cells in the TME has not been clarified clearly. Describing the interactions between various cell compositions and treatments, this review is conducive to constructing BC classifications for survival prediction in the future.

## 2 The cell compositions of the tumor microenvironment

**Figure 1:**
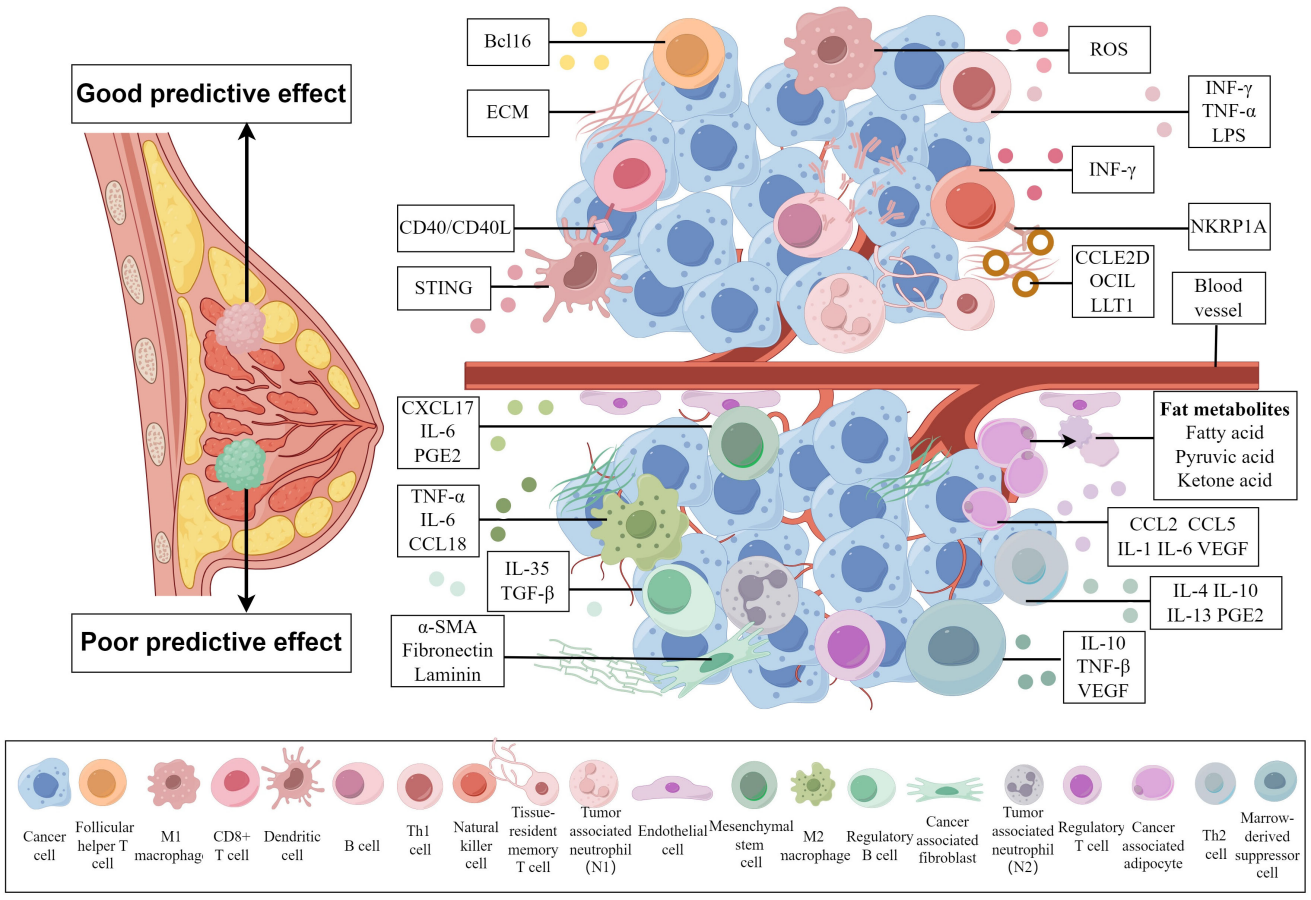
Cell compositions with different predictive effects on the tumor microenvironment of breast cancer and substances related to their effects. A TME above the blood vessels is associated with a good prognosis, while a TME below the blood vessels is associated with a poor prognosis. Different cells influence the prognosis of breast cancer patients by secreting different substances that affect the TME. (By Figdraw).

An increasing number of studies have indicated that the TME plays a major role in the occurrence and treatment effect of BC (12–14). The noncancer cells in the TME of BC include immune cells and stromal cells, whereas the immune cells include T cells, B cells, plasma cells, NK cells, macrophages, myelogenous suppressor cells, dendritic cells and neutrophils. The stromal cells include endothelial cells, pericytes, fibroblasts, adipocytes and mesenchymal stem cells. Different cell compositions play different roles in the treatment of BC. These two types of TME with opposite effects, and the substances associated with cell functions can be observed visually in the **Figure 1**. Research has shown that cells in the TME can be regarded as biomarkers of treatment response. These cells can indirectly reflect the immune status of tumor cells and their hosts, affect tumor development and indicate the pCR after NACT (15). Many comprehensive treatments for BC involve interactions with the TME of the host. Combination administration of targeted cellular compositions of the TME and other conventional treatments has been widely studied, and this approach will be of assistance in follow-up treatment.

### 2.1 Cells with positive predictive therapeutic effects

#### 2.1.1 CD8^+^ T cells

CD8^+^ T cell is one of the vitalest types to predict positive therapy effect. The CD8^+^ T cell is an independent and sensitive predictor of the response to NACT and 5-year disease-free survival (DFS) in patients with BC. Patients with a high percentage of CD8^+^ T cell infiltration are sensitive to NACT (16). The mortality of luminal and HER2-positive BC patients can be predicted by the infiltration of CD8^+^ T cells (3). CAMKK2 can recruit CD8^+^ T cells to accumulate around tumors (17), and HMGB1 can activate the antitumor effect of CD8^+^ T cells to inhibit tumor growth (18). Genome-scale CRISPR screens of CD8^+^ T cells revealed that the interaction between DHX37 and the PDCD11 gene can regulate the NF-κB pathway and the function of CD8^+^ T cells (19). High expression of MAL2 in BC reduces the level and stability of MHC-I on the cell membrane to decrease the cytotoxicity of CD8^+^ T cells and the antigen presentation ability of tumor cells (20). Another study indicated that inhibiting the SOX4 pathway suppresses the appearance of tumor cells whose MHC-I expression are low to improve treatment efficacy (21). The commensal microbiota and their metabolites in the breast can also affect the tumor immune microenvironment. Clostridiales, a kind of commensal microbiota in TNBC, can enhance the antitumor function of CD8^+^ T cells and induce tumor cell pyroptosis by activating the endoplasmic reticulum stress kinase PERK and the related metabolite trimethylamine N-oxide (TMAO) (22). Through various studies, we hope to identify a stable way to promote the proliferation and activation of CD8^+^ T cells in the TME and enhance the immune activity of the TME in BC patients to improve therapeutic efficacy.

#### 2.1.2 Natural killer cells (NK cells)

NK cells are the core cells of innate immunity. NK cells secrete immune-stimulating cytokines and have direct cytotoxic effects on their targets. Immune-stimulating factors enhance the processing and presentation of antibodies. Therefore, these factors can inhibit cell proliferation and angiogenesis, promote cell apoptosis and stimulate the responsive immune system (23). High levels of NK cells are key to achieving PCR after NACT (24). Under the effect of NACT, peripheral NK cells are activated systemically to eliminate metastatic tumors by promoting immune activation and releasing immunosuppressants in the TME (25). Once a tumor occurs, the TME begins to inhibit the function of NK cells (23). In solid tumors, the activity of NK cells in the peripheral blood is poor. In TNBC, NK cells in the peripheral blood can predict the risk of recurrence and progression free survival (PFS) after chemotherapy (26). Lectin-like transcription-1 (such as CLEC2D, OCIL, LLT1) can interact with the NK cell receptor NKRP1A, and blocking this interaction can increase the lytic effect of primary NK cells on TNBC cells (27). It has been reported that blocking A2A receptors can promote NK cell maturation and function, increase the expression of perforin and granzyme, and inhibit tumor metastasis. Tested in clinical trials, A2A/A2B receptor antagonists have potential therapeutic significance in BC (28). Adenosine (Ado) can bind to the A2a and A2b receptors to inhibit the cytotoxicity of CD8^+^ T cells and NK cells. The binding promotes tumor progression and creates an immunosuppressive microenvironment by suppressing inflammatory responses (28, 29). IFN-γ secreted by NK cells can promote the expansion of M1 macrophages, promote the immune activity of the TME (30). In summary, these mechanisms may become new strategies for BC treatment. NK cells play an essential antitumor role in BC. In the future, immunotherapy methods that enhance NK cell function can be combined with conventional antitumor treatments to enhance their efficacy.

#### 2.1.3 M1 macrophages

Tumour-associated macrophages (TAMs) are involved in the innate and adaptive immune responses. In theory, TAMs are differentiated into different functional subgroups under different stimuli, such as cytokines and immune complexes. M1 macrophages are polarized by IFN-γ, TNF-α and LPS secreted by type 1 helper T cells (Th1), and M2 macrophages are polarized by IL-4, IL-10, TGF-β1 and PGE2 produced by type 2 helper T cells (Th2) (8). M1 macrophages can capture, phagocytose and lyse tumor cells, and their antigen presentation ability can promote the cytotoxicity of other immune cells (30). M1 macrophages are associated with a good prognosis in BC patients. Higher levels of macrophages and their gene expression markers are associated with a better NACT response (31, 32). As a classic activation phenotype, M1 macrophages release reactive oxygen species (ROS), reactive nitrogen species (RNS) and proinflammatory cytokines to fight against tumors (8). The release of M1 macrophage-related cytokines leads to a reduction of M2 macrophages in early-stage BC. The expression of M2 macrophages increases when breast cancer progresses and metastasizes (33). Studying the differentiation of TAMs and the relationship between M1 and M2 subtypes are beneficial for the prognosis of BC patients.

#### 2.1.4 Dendritic cells (DCs)

DCs have a key role in initiating and activating adaptive immune responses by presenting antigens and producing specific cytokines. They can interact with T lymphocytes and coordinate T cells activation. DCs are divided into myeloid and plasma-like cell populations based on their surface protein expression. Myeloid dendritic cells (mDCs) take a part mainly in immune cell activation, while plasma-like dendritic cells (pDCs) can produce IFN-I, which is associated with poor prognosis (34). It has been reported that the number of DCs in the TME decreases significantly but the number of DCs in the blood increases after NACT (35). After mature DCs migrate to lymphoid organs, the interaction between CD40 on the surface of DCs and CD40L on T cells delivers antigens to immature T cells in lymphoid organs and activates CD4^+^ helper T cells and CD8^+^ T cells-related cellular immunity (9). The production of interferon and the recruitment of DCs form a part of innate antitumor immunity. A key mediator that promotes DCs to activate T cells is the stimulator of interferon genes complex (STING), which can detect the uptake of tumor cell DNA by host antigen-presenting cells (36). The percentage of DCs in the blood and the expression of human leukocyte antigen DR (HLA-DR), MDC1, CD40 and CD83 on the surface of the primary tumor were significantly reduced after NACT in locally advanced BC. However, the expressions of HLA-DR and CD40 are lower in patients who have a poor response to NACT treatment (35). Cancer cells can inhibit the maturation of tumor-infiltrating DCs, resulting in a weakened ability to present tumor-derived antigens and downregulate the expression of costimulatory molecule (37). Inmmune cells, especially DCs, recruit and activate in the TME when HMGB1 binds to TLR-2, TLR-4, and TLR-9 (38). *In vitro* experiments, DCs equipped with antigens can target tumor cells after being infused into the body. Currently, DC vaccines have been developed and tested in clinical research and are expected to become novel adjuncts for cancer treatment.

#### 2.1.5 Tissue-resident memory T cells (TRMs)

TRM is a kind of new founded cell to predict the positive prognosis of BC. The quantities of studies of TRM have been carried out in recent years. TRMs are the third subtype of memory T cells found in peripheral tissues and intestinal grafts. The other two are central memory T (TCM) and effector memory T (TEM) cells. TCMs reside in lymphoid organs and can be reactivated by secondary infection with the same pathogen. TEMs activate the cytotoxicity of CD8^+^ T cells and patrol in lymphoid and nonlymphoid peripheral tissues (39, 40). CD8^+^ TRM cells are major markers of DFS and OS. TEMs can be used to monitor the immune regulation (41, 42). Single-cell sequencing of the gene of CD8^+^ TRM in BC is significantly associated with improved survival in early TNBC. High-density TRMs are a factor for good prognosis in TNBC patients (41, 43). TRMs can provide rapid and long-term protection at the site of reinfection, and tumor infiltration of TRMs can maintain enhanced therapeutic effects and predict immune therapy responses (44). In the last few years, cancer vaccines and immune checkpoint inhibitors related to TRM, have been suggested to be effective biomarkers for predicting the response of cancer patients to immunotherapy (45). CD103 is a specific marker for TRM cells. TGF-β induces CD103 expression on CD8^+^ T cells to form and maintain TRMs and regulates TRM function through integrin signaling (45). Knocking down Fdft1 or overexpressing PDSS2 can promote the synthesis of coenzyme Q and mitochondrial respiration to facilitate TRM formation and enhance TRM antitumor immunity ability after viral infection (46). We can further study the characteristics of TRMs in the TME to enhance the therapeutic efficacy of BC.

### 2.2 Cells with negative predictive therapeutic effects

#### 2.2.1 M2 macrophages

M2 macrophages are stimulated and polarized by cytokines (such as IL-4, IL-10, IL-13 and TGF-β) from Th2 (47). M2 macrophages are the main TAMs that can form an immunosuppressive TME by releasing cytokines. M2 macrophages promote the occurrence, angiogenesis, invasion and metastasis of BC (48). TAMs can secrete inflammatory cytokines, such as TNF-α, IL-6, and CCL18, promoting the function of BCSCs, the occurrence of EMT, metabolic reprogramming, tumor angiogenesis, and the emergence of therapeutic resistance (49). M2 macrophages can promote tumor invasion and are associated with poor distant metastasis-free survival (DMFS), DFS and OS (50). CD163 is a specific marker of M2 macrophages. High quantities of CD163^+^ TAMs are a predictor of nonmetastatic BC, indicating a poor response to NACT. An increasing infiltration of macrophages in TME indicates an increased risk of recurrence of BC after NACT (51). Macrophage colony stimulating factor (M-CSF) is the main inducer of macrophage migration. By increasing actin and pseudopodial elongation, M2 macrophages are induced to recruit and migrate (52). ROS induce macrophage polarization to the M2 type (53). As a transfer- and inflammation-related microenvironment factor, S100A4 triggers the differentiation and polarization of monocytes into M2 macrophages and increases the secretion of proinflammatory cytokines (54). Increased expression of the NOTCH signaling pathway in endocrine-resistant BC strongly promotes the polarization of TAMs to the M2 macrophages and strengthens endocrine resistance (55). The identification of the molecular mechanisms related to macrophage plasticity and polarization provides the basis for identifying and managing macrophages. Inhibiting TAM infiltration into the TME or polarizing M2 macrophages to M1 macrophages may be potential strategies for treating BC.

### 2.2.2 Marrow-derived suppressor cells (MDSCs)

MDSCs are heterogeneous immature bone marrow cells with potential activity against T cells (56). MDSCs promote regulatory T cells proliferation and inhibit cytotoxic T lymphocytes(CTL) function. The function of MDSCs is to promote proliferation, treatment resistance, angiogenesis, epithelial mesenchymal transition, tumor stemness and metastasis. In addition, MDSCs directly drive tumor growth by reprogramming breast cancer cells (57). Research shows that tumor-infiltrating CD33^+^ MDSC before NACT is a risk factor for tumor progression and the stability of BC, and the possibility of non-PCR. In NACT patients who achieve complete response (CR) or PCR, the number of tumor-infiltrating MDSCs is lower. The lower infiltration of MDSCs is predicting the longer PFS and OS of patients (58). BC can promote the differentiation and recruitment of MDSCs in tumors by secreting cytokines, such as tumor granulocyte colony-stimulating factor (G-CSF), macrophage colony-stimulating factor (GM-CSF) (59), and the chemokine CCL3 (60). IL-10, TNF-β and VEGF secreted by MDSCs are able to activate EMT and angiogenesis, inhibit the immune response, and promote tumor invasion and BC metastasis (61). Therefore, eliminating MDSCs may be a promising therapeutic strategy for preventing immune evasion of BC after surgery.

### 2.2.3 Cancer-associated fibroblasts (CAFs)

CAFs have a prominent role in shaping the TME to support tumor cell survival, metastasis, angiogenesis, immunosuppression and treatment resistance (62). One of the CAF subgroups expressing CD10 supports BCSCs and induces chemotherapy resistance. The higher the expression level of CD10 on CAFs is, the worse the response to NACT will be (63). ECM proteins, such as fibronectin, secreted by CAFs can recruit VEGF1^+^ VLA-4^+^ bone marrow haematopoietic cells into the lung, providing a better and looser environment for metastatic breast cancer cells (64). TGF-β promotes fibroblast proliferation, induces breast cancer cell migration *in vitro*, and promotes the production of αSMA, fibronectin and laminin in CAFs. Inhibiting the differentiation of mesenchymal stem cells to CAFs by blocking TGF signaling weakens their antitumor effects *in vivo* (65). CAFs are associated with maintaining cancer cell stemness. Inhibiting sonic hedgehog signaling with smoothened inhibitors (SMOi) can reduce the expression of BCSCs markers, increase the sensitivity of tumors to docetaxel, reduce the risk of metastasis and increase survival (66). In TNBC patients, high activation of CAFs is positively correlated with lymph node metastasis and infiltration and polarization of M2 macrophages. CAFs may be potential prognostic factors for TNBC accordingly (67). Various subtypes of CAFs have recently been extensively studied in BC. Identifying these subtypes is expected to lead to original approaches for the diagnosis and treatment of BC.

### 2.2.4 Tumor-associated endothelial cells (TECs) and pericytes

TECs are key participants in the growth and invasion of BC. TECs promote tumor angiogenesis and regulate the immune therapy response in the breast TME. Endothelial cells (ECs) transform dormant cancer cells into more invasive and chemoresistant phenotypes (68). ECs activate the Notch signaling pathway to induce neutrophil infiltration and tumor metastasis (69). *In vivo* and *in vitro* experiments have shown that contact between BC and ECs enhances the mesenchymal properties of ECs, which promote the invasion, proliferation and stem cell-like phenotype of BC (70). Pericytes provide survival and structural support for ECs, and their interaction promotes the maturation of the vascular system (71). ECs and pericytes have crucial roles in tumor angiogenesis. Identification of a new target to inhibit the function of ECs and pericytes may reduce tumor angiogenesis and the risk of BC metastasis.

### 2.2.5 Mesenchymal stem cells (MSCs)

MSCs play a crucial regulatory role in various aspects of the pathogenesis of BC. MSCs can promote breast cancer cell proliferation, induce epithelial mesenchymal (EM) transformation of cancer cells, promote the dedifferentiation of cancer cells into tumor stem cells, and promote drug resistance, invasion, immune escape and cancer cell dormancy by secreting molecular mediators (72). IL6R/gp130 signaling in the TME can induce IL-6 secretion by MSCs. The CXCL17 chemokine network can stimulate BCSCs to promote chemotherapy resistance and BC growth (73). Cancer cell-derived IL-6 can trigger prostaglandin E2 (PGE2) production by MSCs, promote bone marrow-derived MCS recruitment to the TME and increase the invasiveness of breast cancer cell (74). TGF-β, the transfer protein Rho-related kinase, matrix protease, and IL-6 are able to activate signaling pathways, such as the MAPK, AKT or WNT pathways, mediating the function of MSCs in promoting BC metastasis (75–77). Therefore, inhibiting the secretion of molecular mediators by MSCs and related pathways limits the growth and progression of BC.

### 2.2.6 Cancer associated adipocytes (CAAs)

Many clinical studies have shown that obesity is related to a high incidence and the poor survival of BC, indicating the fact that adipocytes take a part in the progression of BC (78). CAAs can secrete leptin and the cytokines, such as CCL2, CCL5, IL-1β, IL-6, TNF-α and VEGF to promote the invasion and metastasis of BC (79–81). The interactions between CAAs and breast cancer cells promote CAA to secrete IL-6. In turn, IL-6 can bind to its receptor IL-6R, leading to the proliferation, EMT and metastasis of breast cancer cells (82). IL-6 can also activate the PI3K–AKT axis and HIF-α to increase the production of glucose metabolism, leading to metabolic reprogramming of breast cancer cells, accelerating the rate of glycolysis, and increasing the production of lactic acid (83). In addition, IL-6 can promote ATGL-dependent lipolysis in CAAs (84). It has been reported that CAAs secrete exo-cirCRRIM1 to promote the progression of TNBC (85). Another study showed that CAA upregulates the expression of aromatase in HR-positive BC and promotes the proliferation, metastasis and endocrine resistance of HR-positive BC (86). The decomposition of CAA and fatty acid oxidation products provide breast cancer cells with high-energy metabolites, such as pyruvate, lactic acid, ketone bodies and fatty acids (87). Studying the breakdown metabolism process of CAAs and fatty acid oxidation and the impact of their metabolites on BC is expected to lead to the discovery of novel methods for cancer treatment. For instance, the consumption and decomposition of metabolic products may be a potential way to treat BC.

### 2.3 Cells with unclear predictive therapeutic effects

#### 2.3.1 Regulatory T cells (Tregs)

Tregs are a type of protumor cell that promotes the development of the TME towards a pro-tumor direction and inhibits the cytotoxic effects of T cells and NK cells by producing anti-inflammatory and pro-tumor cytokines. Tregs express immunoregulatory receptors to promote the proliferation, immune escape and metastasis of BC (88, 89). At least two Treg subtypes, including natural CD4^+^ CD25^+^ Treg (nTreg) and inducible Tregs (iTregs) are observed at the tumor site in breast cancer patients. nTreg situated in the thymus can activate immune cells, and iTreg can secrete anti-inflammatory factors (90). Research has shown that low levels of Foxp3^+^ Treg cells in the matrix are significantly associated with improved RFS and OS (91). However, there are also studies indicating that an increase ratio of Foxp3^+^/CD25^+^ tumor infiltrating lymphocytes(TILs) is associated with improved OS (92). A higher Foxp3^+^ Treg cell density is associated with a better prognosis (43). The more Foxp3^+^ Tregs that are consumed, the better the pathological response to neoadjuvant therapy will be (32). Tregs are associated with elevated levels of CD8^+^ T cells, and these favourable outcomes may be attributed to the dominant role of cytotoxic antitumor CD8^+^ T cells in treatment (93). Therefore, the prognostic significance of Tregs in BC is still controversial and needs further study.

#### 2.3.2 B cells and plasma cells

The B-cell lineage, such as CD79a^+^ B cells is infiltrated in almost all patients. Infiltration of B cells enhances local cytotoxic immune response and is positively related to enhancement of the immune status. Plasma cell infiltration in the TME before NACT is related to pCR. In addition, a higher level of PC is related to a better prognosis in hormone receptor-negative BC patients (94). The significant infiltration of B cells in the TME is reportedly associated with improved OS and DFS in TNBC and HER2-positive BC (95, 96). High expression of B cell markers in puncture specimens before NACT is associated with an inproved PCR and patient survival rate (97). However, breast cancer cells induce the production of regulatory B cells (Bregs), which transform resting T cells into Tregs to promote BC metastasis (98). Bregs also produce inhibitory molecules such as PD-L1, FAS ligands, IL-10, IL-35 and TGF-β to inhibit the immune response (99). In conclusion, tumor-infiltrating B cells help to generate effective antitumor immunity at the tumor site, and immunomodulation therapy supporting B cells and inhibiting Bregs may be a promising treatment for BC.

#### 2.3.3 CD4^+^ T cells

CD4^+^ T cells mainly participate in adaptive immunity. Compared with CD8^+^ T cells, the prognostic effect of CD4^+^ T cells is relatively small, and the subgroups of these cells are significantly different. CD4^+^ T cells include Tregs, helper T cells (Th cells), and congenital lymphoid cells (ILCs), which have an influence on host immune regulation. Th cells can be divided into four lineages based on the expression of different genes, named type 1 helper T (Th1) cells, type 2 helper T (Th2) cells, type 17 helper T (Th17) cells and follicular helper T (Tfh) cells. Different lineages have their own unique functions and even opposite functions (100). The different types of CD4^+^ T cells in BC patients determine the malignancy and metastatic capacity of the tumor to a certain extent. INF-γ and IL-12 induce the production of Th1 cells to enhance the ability of antigen-presenting cells to promote the differentiation and clonal expansion of CD8^+^ T cells (101). The combination of immune strategies against Th1 cells and conventional treatments is associated with improved clinical outcomes (102). David G et al. showed that CD4^+^ T cells can enhance the invasion of ECs and the metastatic ability of breast cancer cells by regulating the precancerous characteristics of TAMs. Th2 cells enriched in the TME stimulate epidermal growth factor signaling in breast cancer cells, polarize M1 into M2 macrophages, enhance the pretumor biological activity of myeloid cells, and enhance the intracellular EGF signaling cascade to promote the progression and metastasis of BC (103). Expressing the regulatory factor Bcl6, which is beneficial for antigen-specific B cell maturation, Tfh cells promote local memory cell differentiation, support the development of tertiary lymphoid organs, and have a major role in enhancing the local antitumor immune response (104, 105). However, the role of different subtypes of CD4^+^ T cells in the TME in BC requires further research to develop a targeted drug to promote the differentiation of CD4^+^ T cells into certain subtypes so that the TME can develop into an antitumor state.

#### 2.3.4 Tumor-associated neutrophils (TANs)

Tumor-infiltrating neutrophils (TINs), commonly known as tumor-associated neutrophils (TANs), have two phenotypes. The one is N1 type that has antitumor properties and is highly activated. The another is N2 type that promotes tumor growth, invasion and metastasis and is lowly activated. IFN-γ and IFN-β promote the production of N1-type TANs that promote inflammation and have antitumor effects, while TGF-β induces the production of N2-type TANs that promote inflammation and have antitumor effects (106, 107). Immunocyte deconvolution reveals that the presence of TANs is related to poor prognosis in patients with BC (108). There are also studies indicating that the increased expression of TAN-related genes in TNBC is associated with non-PCR after NACT (97). TANs reduce the proliferation of CD8^+^ T cells in the TME in mouse models of BC and recruit immunosuppressive cells, but their effects on humans have not been determined (109). In summary, there are two subtypes of TANs that have opposite functions. TANs provide predictive information for the therapeutic efficacy of BC treatment and are expected to be useful in the treatment of breast cancer in the future.

## 3 Interaction between conventional treatments and the tumor microenvironment in breast cancer

Breast cancer ranks first in terms of the global incidence rate, but its mortality rate is not high due to the variety of adjuvant treatments. Standard application of adjuvant treatments has greatly improved the prognosis of cancer patients. Different cells in the TME have different roles in treatment. Cells associated with good prognosis can increase the drug sensitivity of tumor cells, and their interaction with therapeutic drugs enhances therapeutic efficacy. Cells associated with poor prognosis can interact with tumor cells to form a feedback loop that enhances the drug resistance of cancer cells or weakens the effect of drugs by secreting cytokines and other substances.

### 3.1 Conventional chemotherapy drugs

Chemotherapy drugs for BC have mainly two mechanisms: one is to interfere with DNA formation, such as anthracyclines, platinum agents and doxorubicin, and the other is to interfere with cell mitosis, such as taxanes (110). The cytotoxicity of chemotherapy drugs come into play by enhancing the functions of cells that predict good prognosis and inhibiting the functions of cells that predict poor prognosis. Research shows that the presence of CD8^+^ T cells during the treatment of BC is related to the improved efficacy of doxorubicin (3). After treatment with doxorubicin and cisplatin, the inflammation-related genes JAK-STAT and TNF-α can be upregulated, which can increase the cytotoxicity of T cell, immune reactivity and the response to PD-1 inhibitors. Short-term treatment with low-dose doxorubicin and cisplatin may result in an immunoreactive TME, increasing the response to PD-1 inhibitors in TNBC patients (111). Paclitaxel enhances the antibody-dependent cell-mediated cytotoxicity (ADCC) effect of trastuzumab by mediating NK cells through NKG2D (112). Paclitaxel induces the secretion of cyclic GMP-AMP synthase (cGSA)-dependent soluble factors in breast cancer cells and promotes the polarization of TAMs into M1 macrophages (113). Regulated by paclitaxel and docetaxel, the number of MDSCs decreases, but the number of M1 macrophages remains the same. Docetaxel promotes the differentiation of MDSCs into M1 macrophages (114). Commonly used chemical and targeted drugs, such as taxanes, anthracyclines, and anti-HER2 monoclonal antibodies, can directly induce immune stimulation to kill tumor cells by activating DCs (14).

Chemotherapy drugs, such as taxanes and anthracyclines, can kill “bad” lymphocytes such as Tregs and cause immunogenic cell death (ICD), and help restore antitumor immunity (115, 116). It has been reported that, compared to single therapy, combination administration of cisplatin and inhibition of Tregs migration enhances the anticancer effect (117). Combination administration can reduce the recruitment of TAMs and downregulate the expression of chemotherapy resistance genes and multidrug resistance genes. For example, CSF-1 can promote the recruitment of TAMs, and the combination of anti-CSF-1 therapy and chemotherapy drugs can reverse chemotherapy resistance (118). Chemotherapy often leads to the occurrence of chemotherapy resistance and severe systemic toxic reactions that impair immune function. Adriamycin and cisplatin promote macrophage infiltration by increasing the expression of CCR2 in myeloid cells, leading to therapeutic resistance (119). LINC00337 accelerates malignant characteristics and promotes paclitaxel chemoresistance in breast cancer cells through M2 macrophages (120). M2 macrophages secrete high levels of IL-10 and induce the recruitment of drug-resistant TAMs through the IL-10/STAT/Bcl-2 signaling pathway. Paclitaxel, etoposide, and doxorubicin induce M2 macrophages to secrete tissue proteases and induce tumor cell death (121, 122). M2 macrophages and CSCs participate in gap junctional intercellular communication (GJIC), which causes carboplatin resistance in BC (123).

Chemotherapy-resistant breast cancer cells can promote the proliferation of stroma cells associated with poor prognosis and promote the survival of chemotherapy-resistant cancer cells. Doxorubicin-resistant 4T1 cells release IL-33 to promote MDSC accumulation and reduce doxorubicin-induced tumor cell death (124). Doxorubicin-polyglycerol-nanodiamond conjugate (Nano-DOX) has been shown to downregulate tumor-derived granulocyte colony-stimulating factor (G-CSF) and inhibit the induction and infiltration of MDSCs. Nano DOX induces the release of damage-associated molecular patterns (DAMPs) in 4T1 cells, stimulating the tumor immune microenvironment by activating key antitumor immune cells, such as macrophages and DCs (125). Research has shown that ECs exposed to chemotherapy secrete TNF-α, activate NF-κB signaling pathway and promote CXCL1/2 expression, leading to amplification of the CXCL1/2-S100A8/9 loop and inducing doxorubicin resistance (126). CAFs mediate chemotherapy resistance by releasing collagen I, which can reduce the absorption of drugs by cancer cells. Docetaxel-induced overexpression of MMP-1 and collagen VI in CAFs can protect breast cancer cells from docetaxel-induced cell death. The research indicates that CAFs and the collagen secreted by them are potential targets for the treatment of BC (127, 128). Armornsupak et al. reported that CAFs can increase HMGB1 expression in breast cancer cells and promote doxorubicin resistance (129). It has been reported that MSC induces chemotherapy resistance (such as doxorubicin and 5-fluorouracil) via a CD9-dependent mechanism in BC and enhances the expression of drug-resistant proteins (BCRP and MDR1). MSC-CD9 may become an important target for the treatment of BC (130). Adriamycin and paclitaxel promote the production of the serine enzymes E1, CCL2, IL-6 and IL-8 in MSCs and promote breast cancer cell proliferation and angiogenesis (131). Adriamycin can also cause MSCs to produce miR-21-5p to lead to chemotherapy resistance. The miR-21-5p is delivered to adjacent tumor cells through exosomes (132). After co-culture of breast cancer cells and adipocytes, the expression of MVP is upregulated in BC to decrease the intake of chemotherapeutic drugs and reduce doxorubicin resistance (133).

**Figure 2:**
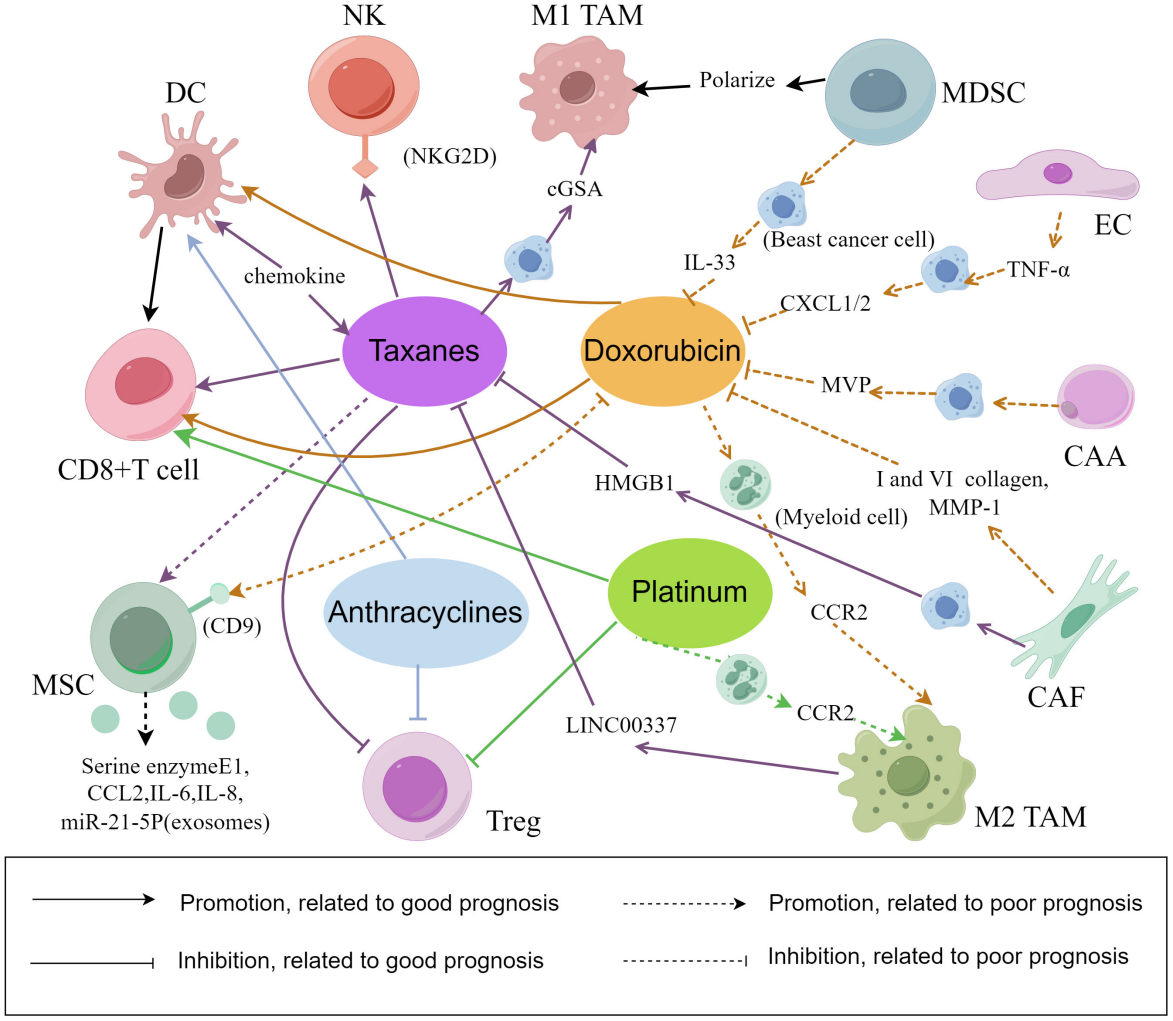
The interaction between chemotherapy and cell composition in the tumor microenvironment. There are several mechanisms underlying the relationship between chemotherapy efficacy and the cellular composition of the TME in breast cancer. Some mechanisms can promote breast cancer cell lysis and apoptosis, while others can lead to breast cancer cell proliferation, epithelial mesenchymal transformation and chemotherapy resistance. (By Figdraw).

The mechanisms of cells in the TME during the chemotherapy are shown in the **Figure 2**. The combination of regulating the number of different cells associated with prognosis and chemotherapy significantly reduces primary tumor progression, reduces metastasis. In summary, the antitumor TME can be reprogrammed and formed to enhance the response to cytotoxic therapy.

### 3.2 Antitumor molecular targeted therapies

HER2-positive BC is often treated with chemotherapy and anti-HER2 targeted therapies, mainly trastuzumab and patuzumab. The first mechanism of these two targeted drugs is inhibiting HER2-mediated cellular signal transduction (134). The second is utilizing NK-cells- and T-cell-mediated ADCC, and macrophage-mediated antibody-dependent cellular phagocytosis (ADCP) to eliminate cancer cells (135). In addition to their own anti-tumor mechanisms, trastuzumab and pertuzumab can also reshape the TME, which is correlated to the efficacy of anti-HER2. Trastuzumab downregulates CCL2 and increases the expression of PD-1 in HER2-positive BC, thus increasing the immune index and Tfh cells infiltration (136). Trastuzumab and pertuzumab bind to HER2 receptors on the cancer surface through FcγR connections, promoting NK cell recruitment around cancer cells and prolonging the cytotoxicity of chemotherapy (137). After the use of trastuzumab for NACT, trastuzumab increases the ADCC effect on patients’ immune cells cultured *in vitro* (138). During NACT, trastuzumab mediates the ADCP effect on TAMs and results in immunosuppression. B7-H4 in the TAM of HER2-positive BC patients is significantly upregulated, leading to immune escape of breast cancer cells (139). Contacting breast cancer cells with MSCs activates src kinase to downregulate the expression of the PTEN gene and activate the PI3K/AKT signaling pathway, causing trastuzumab resistance (13). MSCs induce the expression of the lncRNA AGAP2-AS1 in breast cancer cells, promote the expression of CPT1, induce FAO to induce tumor cell stemness, and lead to trastuzumab resistance (140). CAA induces trastuzumab resistance by activating the IL-6 or STAT-CPT1B-FAO axis (141). These studies identified potentially useful biomarkers for HER2-positive BC.

The other anti-HER2 targeted therapies also have the interaction with cells in the TME. IL-8 activates the Scr/STAT/ERK1/2 pathway and mediates EGFR signal transduction. TAMs secrete IL-8 in the TME of locally advanced BC, leading to HER2-positive BC resistance to lapatinib (one kind of TKI) and trastuzumab (142).. Marusyk et al. reported that fibroblasts can inhibit lapatinib-induced cell death in a variety of breast cancer cell lines and increase the apoptosis threshold of breast cancer cells. The combination administration of BCL2/BCL-xL inhibitors and lapatinib can restore the apoptosis sensitivity of some breast cancer cells (143). CAA increases glycolysis in BC, which leads to an increase ratio of lactic acid/glucose in HER2-positive BC patients and reduces the secretion of interferon-γ by NK cells, causing resistance to lapatinib and trastuzumab (144). Therefore, many cells in the TME are involved in anti-HER2 targeted therapy. Studying and regulating these cells and cell products may reverse treatment resistance, which is conducive to the survival and prognosis of HER2-positive BC patients.

In the past few years, many original therapeutic targets for malignant tumors have been discovered. The interaction of these novel targeted therapies with cells in the TME has been studied extensively. High macrophage infiltration activates the NF-κB signaling pathway through IL-6, leading to resistance to PI3K inhibitors, hedgehog inhibitors (Cyclopamine), and BET inhibitors (145–147). With the sustained influence of bevacizumab, TAMs polarize into the M2b subtype. With the increasing proportion of M2 macrophages, TNF-α produced by them promotes IDO1 expression in cancer cells and leads to bevacizumab resistance (148). In the treatment of BC, PARP inhibitors can activate the cGAS/STING pathway in tumor cells and induce the recruitment and activation of CD8^+^ T cells and DCs (149). Olaparib, the PARP inhibitor can control the state, phenotype, function, and metabolism of TAMs, increasing recruitment of TAMs to tumors. The BC with olaparib resistance remodels glucose and lipid metabolism in TAMs through sterol regulatory element binding protein 1 (SREBP1). A PARP inhibitor combined with an anti-CSF-1R antibody can enhance CD8^+^ T-cell-mediated innate and adaptive antitumor immunity and prolong the survival of breast cancer patients with the BRCA gene mutation (150). Research has shown that the expression of the chemokine receptor gene CCR8 is upregulated in tumor-resident Tregs compared with that in normal tissue-resident Tregs. Targeting CCR8 to clear Tregs in the TME may be a promising immunotherapy method for BC (151). Cetuximab, an IgG1 monoclonal antibody targeting EGFR, can trigger the ADCC of TNBC cells by NK cells, but this ability is negatively correlated with the expression of CD85j. CD85j antagonists can restore the ADCC effect and improve the clinical efficacy of cetuximab (152). The protein tyrosine phosphatases PTPN1 and PTPN2 are core regulatory factors of inflammation, and these gene deletion in tumor or immune cells can promote antitumor immunity (153). ABBV-CLS-484 (AC484), an effective inhibitor of PTPN1 and PTPN2, activates the JAK/STAT pathway, reduces T-cell dysfunction, stimulates inflammatory responses in the TME, and promotes the function of NK cells and CD8^+^ T cells (154). Biodegradable polymer and zinc phthalocyanine photosensitizer-NPs target the mitochondria of cancer cells and induce DC activation and tumor antigen generation through light activation (155).

In summary, commonly used antitumor molecular targeted therapies combined with targeted cells in the TME therapy can induce persistent reprogramming of the TME, which can constitute a promising treatment strategy for BC.

### 3.3 Endocrine therapy

Endocrine therapy resistance is associated with inhibition of cells that are associated with good prognosis and activation of cells that are associated with negative prognosis in the TME of HR-positive BC. Oestrogen can regulate the activity and metabolism of different subtypes of macrophages and T cells. According to previous research, oestrogen can slow the apoptosis of neutrophils, leading to a decrease in the cytotoxic activity of NK cells. Inhibiting oestrogen can increase the activity of circulating NK cells and reduce the infiltration of Tregs into the TME (156, 157). In CD4^+^ T cells, oestrogen signaling is associated with inhibiting Th1 cells to secrete proinflammatory cytokines and promoting Th2 cells to produce anti-inflammatory cytokines (158).

It has been reported that Foxp3^+^ Tregs are associated with poor survival in patients with HR-positive BC (159). Increased oestradiol in the breast promotes the polarization of monocytes into M2 macrophages, the differentiation of CD4^+^ T cells into Tregs and the infiltration of Tregs and MDSCs. These mechanisms suggest that oestradiol inhibits the formation of antitumor TME (12, 160). Research shows that tamoxifen resistance is related to high EGFR expression, and the quantity of TAMs infiltrated in BC tissues with high EGFR expression is large. TAMs can synthesize various cytokines to enhance aromatase activity and increase oestrogen production (161, 162). CAAs activate the JAK/STAT pathway, increase leptin secretion, lead to Hsp90 expression, and reduce sensitivity to tamoxifen (163). The expression of integrin-β1 in breast cancer cells induced by CAFs can promote the proliferation and drug resistance of dormant cancer cells after treating with fulvestrant (164). Blocking aromatase to reduce oestrogen synthesis by targeting cells in the TME may provide a new therapeutic way in HR-positive BC.

### 3.4 Radiotherapy

Radiotherapy is a fundamental method for treating BC and has prominent antitumor effects under the effect of cells in the TME. Research shows that the therapeutic effect of breast-conserving surgery plus radiotherapy is equivalent to that of total mastectomy. After treatment with breast-conserving surgery, radiotherapy can reduce mortality and recurrence (5). The death of tumor cells caused by radiotherapy can stimulate the activity of DCs and CTLs to kill cancer cells (165). Radiotherapy can induce the expression of CXCL16, which is a proinflammatory cytokine that enhances the recruitment and antitumor cytotoxicity of CD8^+^ T cells (166). Radiotherapy can induce cancer cells damage or death, leading to the release of damage-associated molecular patterns (DAMPs), including ATP, HMGB1, calreticulin, and heat shock proteins (167). HMGB1 can activate Toll-like receptor 4 on DCs, enhance the cross-presentation of tumor antigens, and subsequently activate the response of CTLs (14, 168). Radiotherapy at one site can activate DC migration and cross-activate T cells in lymph nodes to induce systemic antitumor immunity (abscopal effect). The effect helps eliminate tumors in nonradiation-treated metastatic sites (169).

Radiotherapy can promote BC progression through interaction with cells that are associated with negative prognosis in the TME. After radiotherapy, breast cancer cells produce CCL2, which stimulates TAM recruitment. TAMs further induce angiogenesis and secrete immunosuppressive factors such as IL-10 and TNF-β to promote radiation tolerance and tumor metastasis (170). Radiotherapy can upregulate the expression of IL-1P, TNF-α, IFN-I, and IFN-II and induce the expression of cell adhesion factors (such as VCAM-1 and ICAM-1) on ECs, which promote lymphocyte migration to the tumor parenchyma (171, 172). Radiation-induced DNA damage can increase the activity of the NF-κB signaling pathway in ECs and promote the production of IL-6, CCL1, and CCL5 (173). These cytokines can attract Tregs and maintain the protumor TME (174). CAFs protect BC from the impact of radiotherapy by releasing exosomes to activate NOTCH3- and STAT1-dependent pathways. CAF-mediated drug resistance can be prevented through combined with γ-secretase inhibitors (175). During radiotherapy for breast cancer patients, combination administration of drugs targeted cell compositions together can enhance the effect of radiotherapy after detecting the cellular compositions of the TME.

### 3.5 Immunotherapy

Immunotherapy can effectively inhibit the development of BC. The main immune checkpoint inhibitors in BC treatment include PD-1/PD-L1, TIM-3, and CTLA-4 inhibitors, which can present tumor-related antigens to T lymphocytes through DCs and enhance the antitumor activity of CD8^+^ T cells (9). It has been reported that neither anti-PD-L1 therapy nor anti-CD73 therapy alone significantly inhibits tumors in xenograft mice, but combination therapy can inhibit tumor growth by increasing the number of CD8^+^ T cells and the ability to secrete INF-γ and TNF-α (6). Avelumab is a human anti-PD-L1 IgG monoclonal antibody whose mechanism is not to block the PD-1/PD-L1 signaling pathway but to trigger the ADCC effect in TNBC. The ADCC is activated by IL-2 and IL-15, which can enhance NK cell-mediated cytotoxicity (176). CD8^+^TRMs are biomarkers for predicting the response to ICI immunosuppressive therapy. Patients with advanced BC and high quantity of TRMs have a greater response to PD-1 inhibitors. Blocking the PD-1/PD-L1 interaction can increase the toxicity of CD8^+^ TRMs to cancer cells (44). After blocking TIM-3 with an anti-(α)TIM-3 antibody, the chemokine released by DCs increases to enhance the function of T cells and the response to chemotherapy. The use of an anti-TIM-3 antibody can especially improve the response to paclitaxel in luminal B BC and TNBC in mouse models (177).

A study in TNBC showed that the combination of phosphoinositol 3 kinase δ (PI3Kδ) inhibitors and PD-1 inhibitors can increase the proportion of CD8^+^ T cells, reduce the number of Tregs and MDSCs, and improve the efficacy of radiotherapy (178). High expression of TYRO3 in BC can reduce the M1/M2 macrophages ratio, inhibit iron death, generate a promoting TME, and lead to resistance to PD-L1 inhibitors (179). Cytokines derived from MSCs, such as CCL5, can lead to the upregulation of PD-L1 in BC. Inhibiting MSCs with the cytokine inhibitor pirfenidone can significantly reduce the secretion of CCL5 in breast cancer cells to reduce the expression of PD-L1 (180). PD-L1 expressed in CAA can inhibit the antitumor function of CD8^+^ T cells activated by anti-PD-L1 antibody, leading to resistance to PD-L1 inhibitors. The PPARγ antagonist GW9662 can enhance the antitumor effect of CD8^+^ T cells. It can also selectively inhibit the expression of PD-L1 in mouse adipose tissue and the progression and metastasis of BC (181). PD-L1 inhibitors and FOXP3^+^ Tregs may have synergistic effects. The upregulated expression of these genes in the TME can promote tumor immune evasion in BC. The combination of PD-L1 inhibitors and consuming Tregs can improve the therapeutic efficacy in TNBC patients (88). TINs enriched in the TME help breast cancer cells escape ferroptosis and become resistant to immune checkpoint inhibitors. Aconitate decarboxylase 1 (Acod1) is highly expressed in human TIN. Downregulation of Acod1 can reduce the infiltration and immunoregulation of TIN, inhibit the metastasis of BC, and increase the efficacy of immune checkpoint inhibitors (182). Receptor tyrosine kinase RON expressed by macrophages inhibits the tumor immune response, promotes the binding of CD80 to CTLA-4, inhibits T-cell activation, and leads to resistance to CTLA-4 inhibitors (183). The study of overcoming immunotherapy resistance by targeting cells with a poor prognosis in the TME has prospective clinical significance and will greatly improve the efficacy of immunotherapy in BC patients.

### 3.6 Bisphosphate

Bisphosphate is used to treat bone metastases and improve its own efficacy by affecting cell compositions in the TME. Zoledronic acid is one kind of bisphosphate that can reduce the expression of MMP-9 on TAMs, increase the number of M1 macrophages, and increase the survival rate of breast cancer patients. Zoledronic acid can also prevent MSCs from producing MCP-1 to recruit TAMs to tumors and control tumor growth (184).

Altogether, cells in the TME have rich mechanisms of action in treatment. There are interactions between different cellular compositions and between cells and therapy. Some cells provide physical and mechanistic support for cancer cells to promote tumor progression, while others lyse and kill cancer cells to inhibit tumor progression. **Table 1** summarizes the commonly associated cells and their main mechanisms of each conventional treatments.

**Table 1:** Different roles of cells in the tumor microenvironment during treatment.

Treatment		The effect of related cells	Main Mechanisms	References
Chemotherapy	Paclitaxel	NK, M1	Promote therapeutic effects, Promote TAM polarization to M1	(111–113)
		Treg, MDSC, CAF, M2	Immunogenic cell death, Promote cancer cell proliferation, Development of treatment resistance	(115, 120, 121, 124, 127)
	Doxorubicin	CD8^+^ T, DC	Promote therapeutic effects, Activate DC function	(3, 14, 111)
		MDSC, CAA, CAF, MSC, M2	Promote cancer cell proliferation, Reduce the effect of chemotherapy, Develop treatment resistance	(122, 124, 129, 130, 133)
	Platinum	CD8^+^ T	Promote therapeutic effects	(111)
		Treg, M2	Immunogenic cell death, Develop treatment resistance	(115, 119)
Targeted antitumor drugs	Trastuzumab	NK	ADCC	(135)
		MSC, CAA	Develop treatment resistance	(13, 140, 141)
	Lapatinib	TAM, CAF, CAA	Develop treatment resistance, Increase glycolysis	(142–144)
	Bevacizumab	M2	Promote TAM polarization to M2	(148)
	PARP inhibitor	CD8^+^ T, DC	Promote immune cell recruitment	(149)
		TAM	Promote TAM glucose metabolism and lipid metabolism	(150)
Endocrine inhibiting drugs	Tamoxifen	TAM, CAA	Promote oestrogen production, Reduce drug sensitivity	(161, 163)
	Fulvestrant	CAF	Promote proliferation and treatment resistance	(163)
Radiotherapy		DC, CD8^+^ T	Promote immune cell activity and recruitment	(165, 166)
		TAM, EC, Treg, CAF	Induce angiogenesis, chemokines and cytokines	(170–175)
PD-1/PD-L1 Inhibitory		NK, TRM	ADCC, Promote therapeutic effects	(44, 176)
		CAA, Treg	Develop treatment resistance, Promote immune escape of cancer cells	(88, 182)
Zoledronic acid		M1, MSC	Promote TAM polarize to M1, Control tumor growth	(184)

## 4 Targeting the cellular composition of the tumor microenvironment to treat breast cancer

With the arrival of the era of precision treatment for BC, the classification of BC based on the different roles of cells in the TME has been widely studied. In the future, it will be necessary to investigate these studies in clinical trials to determine the effects of various cell compositions in the TME on patient survival and prognosis. The combination of conventional first-line and second-line therapies with drugs targeting the cells in the TME is a promising approach to achieve better therapeutic effects and represents a new direction for future research.

**Figure 3:**
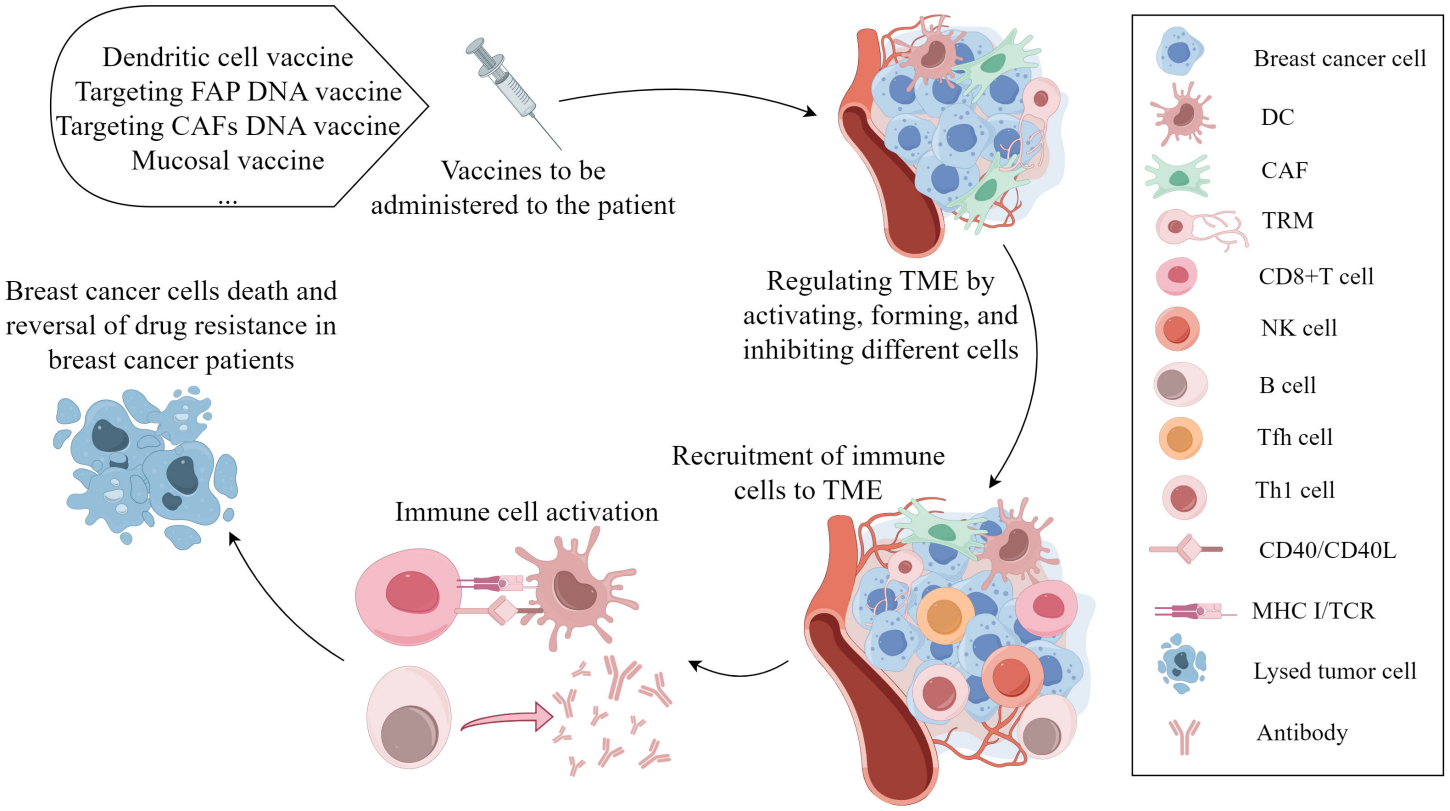
The mechanism of vaccines targeting cell compositions in the tumor microenvironment in the occurrence and progression of breast cancer. Vaccination leads to various effects, such as DC activation, TRM formation, and CAF function inhibition. These antigen-presenting cells cross-present tumor-related antigens to cytotoxic CD8^+^ T cells, activating CD8^+^ T cells, B cells, and NK cells to induce tumor cell death and reverse chemotherapy resistance. (By Figdraw).

CD8^+^ T cells and NK cells have significant roles in the efficacy of cancer vaccines. The vaccines can specifically amplify and activate CD8^+^ T cells and NK cells, but their effectiveness in controlling cancer development is limited (185). A dendritic cell vaccine (DCV) combined with NACT can improve the pCR and ameliorate PD-L1-negative tumors. DCV can reshape the TME and induce cellular and humoral immunity in peripheral blood (186). In the phase I clinical study, before surgical resection, the vaccine containing the HER2 peptide was directly injected into the lymph nodes of BC patients. The results showed that the vaccinated patients developed antigen-specific immunity and exhibited high levels of HER2-specific CD4^+^ Th and CD8^+^ T cells. In addition, DC vaccines with the HER2 peptide lead to lymphocyte accumulation in the breast and induce ADCC. DC vaccines may prevent and treat early BC (187). Local administration of mucosal vaccines can promote the formation of TRMs, which are biomarkers of cancer vaccine response and immune response (45). The expression of cancer fibroblast activator protein (FAP) in CAFs is upregulated. Loeffler, M. et al. developed a DNA vaccine targeting FAP, which can target CAFs in BC, reverse the chemotherapy resistance, and reduce the growth and metastasis (188, 189). According to previous reports, the anti-FAP vaccine can reduce the accumulation of type I collagen and increase the uptake rate of docetaxel by tumors by 70% (188). A novel DNA vaccine targeting CAFs can enhance the antitumor metastasis function of doxorubicin, inhibit the expression of the IL-4 and IL-8, and promote the recruitment of CD8^+^ T and DCs (190). In conclusion, cancer vaccines that act on the TME are being extensively studied and have gradually attracted increasing amounts of attention in the treatment of BC. The process of vaccines targeting cell compositions in the TME is clearly present in the **Figure 3**.

Metformin is used to treat patients with type 2 diabetes and has recently been found to reduce the incidence of cancer. Teufelsbauer et al. reported that, when treated with metformin, MSCs preferentially differentiate into the osteogenic lineage, thus releasing factors that inhibit the migration of breast cancer cells (191). The exosomal CXCR4^+^ TRAIL is transduced from MSCs via a lentiviral vector. These exosomals promote MSC homing and induce cancer cell apoptosis. These Exosomes produced by MSCs can pass through the blood–brain barrier, cooperate with the antitumor effect of carboplatin, and shrink brain metastatic lesions in patients with BC (192). It has been reported that the bioactive compound sulforaphane from broccoli can inhibit the occurrence and growth of BC, and prevent the differentiation of fat cells and the interaction with BC (193). Metformin, the renin–angiotensin inhibitor, aspirin and EGCG, a component of green tea extract, can inhibit the differentiation of adipose-derived stem cells (ADSCs) into adipocytes. These substances can also limit the proliferation and invasion of breast cancer cells (194–197). Aspirin can inhibit adipogenesis and oxidative stress to change the metabonomics and fatty acid composition of adipocytes. Under the effect of aspirin, obesity-related inflammation and the growth and migration of BC are suppressed (197). These drugs can directly or indirectly affect cells in the TME to reduce tumor proliferation and progression. In-depth study of the role of these drugs in the TME may be useful for improving the prognosis of BC patients in the future.

## 5 Discussion

The cell compositions of the TME play a complex role in the breast TME and have a regulatory impact on the whole process of tumor occurrence. There are more and more evidences that the cellular compositions of TME have a profound influence during the treatment of BC. The combination administration of targeted cells in the TME and conventional therapy is a feasible method for BC treatment in the future. In the TME, cells associated with a good prognosis include CD8^+^ T cells, NK cells, M1 macrophages, DCs and TRMs (8, 16, 23, 34, 41). They secrete tumor-inhibiting factor and immune-stimulating cytokines which are able to kill cancer cells, inhibit tumor angiogenesis and activate the immune system to prevent tumor growth and metastasis. For another, cells in the TME associated with a poor prognosis include M2 macrophages, MDSCs, CAFs, TECs and pericytes, MSCs and CAAs (48, 56, 62, 68, 71, 72, 78). They secrete cytokines which are able to promote tumor angiogenesis, treatment resistance to promote tumor occurance and progression. In addition, there are some cell compositions whose prediction effect are unclear, including Tregs, B cells, CD4^+^ T cells and TANs (88, 98, 100, 106). Therefore, the function of cells in the TME deserves advanced study. There are also interactions between cells, for instance, NK cells promote activation of CD8^+^ T cells and polarization of M2 macrophages (29, 30). Different subtypes of the same cell have different functions, such as macrophages, CD4^+^ T cells, TANs and DCs (8, 34, 100, 106). It is a possible treatment approach that promote cell polarization toward to certain subtypes that are positive to the prognosis of BC patients.

The mechanism of cells in the TME manifests as a direct effect on cancer cells or an indirect impact on the TME. Under the influence of the differences in cellular compositions in TME, therapeutic effect is different in BC patients after therapy. In the meantime, the cellular composions in TME are changed. These changes predict the prognosis including OS, DFS and PFS of BC patients. All the treatments have two opposite effects on the cell compositions in the TME. The good one is associated with apoptisis, and the bad one is related to treatment resistance (12, 111, 144, 171, 178). As vaccines, lentiviral vectors, non-anticancer drugs and biological extracts were found to have positive implications for cellular compositions changes in the TME, we see the benefits of combination administration targeted cellular compositions of the TME and other conventional treatments in BC (187, 191, 193, 197). The cellular compositions of the TME and and therapeutic mechanisms deserves further study.

## 6 Conclusion

In recent years, “immune hot” and “immune cold” tomours are discussed extensively. But in the TME, the individual role of each cell has not been comprehensively elaborated yet. We synthesize a lot of research and indicate that the TME plays a crucial role in the development and treatment of BC. This review details the main cell compositions in the TME of BC and their possible differential effects on the occurrence, development and treatment of BC. It’s worth noting that targeting the cellular compositions of the TME is promising to be used for combination administration. As far as we know, targeting some substances such as cytokines will affect certain specific cells in the TME to help establish an antitumor TME and promote the progression of BC treatment. In future research, we will combine drugs targeting specific cells in the TME with conventional drugs for BC treatment to improve the efficacy of these drugs and reduce drug resistance and tumor recurrence. In future research on the treatment for BC, the influence of conventional therapy (such as chemotherapy, radiotherapy and targeted therapy) on the host antitumor immune response, the host-versus-host response (autoimmunity toxicity) and cell composition in the TME must be considered. We may be able to treat BC patients more accurately according to differences in the cell compositions of the TME. Precise treatment can be achieved by remodelling the TME, promoting antitumor cell function, inhibiting tumor-promoting cell function, researching a cell compositions vaccine for the TME, and combining drugs that affect signaling pathways.

In conclusion, various cell compositions in the TME play crucial roles in regulating breast cancer and therapeutic effects. A better understanding of the TME will provide a new direction for exploring new and effective precision treatment schemes for breast cancer.

## Author contributions

TD: Conceptualization, Data curation, Methodology, Writing – original draft. JL: Conceptualization, Data curation, Writing – original draft. YZ: Conceptualization, Data curation, Writing – original draft, Visualization. WP: Conceptualization, Data curation, Writing – original draft. ZB: Conceptualization, Writing – original draft, Methodology. BW: Methodology, Writing – review & editing. YW: Writing – review & editing, Conceptualization, Funding acquisition. JH: Conceptualization, Funding acquisition, Writing – review & editing.
